# Intraspecies variation of the mitochondrial genome: An evaluation for phylogenetic approaches based on the conventional choices of genes and segments on mitogenome

**DOI:** 10.1371/journal.pone.0273330

**Published:** 2022-08-18

**Authors:** Jesús Morón-López, Karen Vergara, Masanao Sato, Gonzalo Gajardo, Shoko Ueki

**Affiliations:** 1 Institute of Plant Science and Resources, Okayama University, Kurashiki city, Okayama, Japan; 2 Laboratorio de Genética, Acuicultura & Biodiversidad, Departamento de Ciencias Biológicas y Biodiversidad, Universidad de Los Lagos, Avda, Osorno, Chile; 3 Division of Applied Bioscience, Graduate School of Agriculture, Hokkaido University, Sapporo, Hokkaido, Japan; Sichuan University, CHINA

## Abstract

Intraspecies nucleotide sequence variation is a key to understanding the evolutionary history of a species, such as the geographic distribution and population structure. To date, numerous phylogenetic and population genetics studies have been conducted based on the sequences of a gene or an intergenic region on the mitochondrial genome (mtDNA), such as cytochrome c oxidase subunits or the D-loop. To evaluate the credibility of the usage of such ‘classic’ markers, we compared the phylogenetic inferences based on the analyses of the partial and entire mtDNA sequences. Importantly, the phylogenetic reconstruction based on the short marker sequences did not necessarily reproduce the tree topologies based on the analyses of the entire mtDNA. In addition, analyses on the datasets of various organisms revealed that the analyses based on the classic markers yielded phylogenetic trees with poor confidence in all tested cases compared to the results based on full-length mtDNA. These results demonstrated that phylogenetic analyses based on complete mtDNA sequences yield more insightful results compared to those based on mitochondrial genes and segments. To ameliorate the shortcomings of the classic markers, we identified a segment of mtDNA that may be used as an ‘approximate marker’ to closely reproduce the phylogenetic inference obtained from the entire mtDNA in the case of mammalian species, which can be utilized to design amplicon-seq-based studies. Our study demonstrates the importance of the choice of mitochondrial markers for phylogenetic analyses and proposes a novel approach to choosing appropriate markers for mammalian mtDNA that reproduces the phylogenetic inferences obtained from full-length mtDNA.

## Introduction

Mitochondria, the organelles present in most contemporary eukaryotic organisms, are considered cellular power plants due to their essential role in energy production. It has been well established that modern mitochondria originated from a bacterial endosymbiont, which probably belongs to an α-proteobacteria ancestor, in a proto-eukaryotic host [1].

As a remnant of being a symbiotic organism, mitochondria possess a genome (mtDNA) that codes for the part of the proteins constituting the organelle. Owing to its evolutionary history as an endosymbiont transformed into innate cellular machinery, the ancestral genome has been substantially altered and reduced into contemporary mtDNA, and many of the original genes have been transferred to the nucleus or lost [2,3]. Consequently, mtDNA genome diversity, with a variety of structures, sizes, and gene contents, emerged across the Eukaryota over the course of evolution. For example, in Metazoa, mtDNAs are circular and relatively small compared to other taxa [4–6]. Generally, metazoan mitochondrial genes lack introns, while a single large noncoding segment, the control region or the D-loop, is found in bilaterian animals [7,8]. The mtDNA typically contains 13 protein-coding genes that code for the electron transport chain and oxidative phosphorylation, 22 tRNA genes, and the genes that code for the large and small rRNA subunits. Gene content, organization, size, translation codes, and uniparental inheritance are preserved among most of the metazoan [4–6], with some exceptions in non-bilaterianism [9]. While animal mtDNA are as small as 15 to 17 kb, plant mtDNAs are much larger [10,11]: angiosperm mtDNAs are typically in the range of 200 to 700 kb, with an extreme example, as large as 11 Mb, found in *Silene conica* [12]. Fungi mtDNA have been less explored compared to their animal and plant counterparts [13]. Most of the fungi mtDNA studied to date are circular, but there are a few species possessing linear forms [14]. Fungi mtDNAs resemble those of plants in certain features, for example, the presence of a variable number of groups I and II large introns and intergenic regions [15,16], while the kingdom is often associated with Metazoa. The presence of mobile endonuclease open reading frames (ORFs) in these introns represents one of the major sources of variability in fungal mtDNA [17]. In the case of ‘catch-all kingdom’ protists, their mtDNA is known to be diverse [18]. For example, the simplest form of mtDNA is found in myxozoans, alveolates, which encode only subunits 1 and 3 of cytochrome c oxidase (cox), one subunit of cytochrome c reductase (*cob*), and short, functional fragments of small and large subunits of mitoribosomal RNA [19–21]. In contrast, the most gene-rich mtDNA identified to date belongs to jakobids [22]. The structures of protist mtDNAs are also diverse: while many protists possess a circular or a permutated linear, some dinoflagellate mtDNAs consist of small linear fragments that contain many pseudogenes and many non-functional gene fragments [23–26].

Existing in virtually all eukaryotic organisms and, due to their small sizes in comparison to chromosomes, their conserved genetic components and fast evolutionary rate [5,6,27], mtDNA has been regarded as an excellent marker for phylogeny, phylogenetics, and population structure studies for decades. For example, the non-coding region D-loop in animals is adopted as a marker due to its sequence variation. For animals, fungi, plants, and protists, some well-known core mitochondrial genes, such as *cox1*, *cob*, or *nad6*, have been frequently used to study the speciation processes as well as intraspecific genetic variations. This is because these genes are longer than other mitogenes, and longer sequences presumably better represent the phylogeny of the entire mtDNA. However, several studies have demonstrated that the phylogenetic inferences obtained from the entire mtDNA and the partial sequences, both D-loop and protein-coding sequences, may be discordant in some cases [28–30]. In addition, we identified two hypervariable genes on the mtDNA of a protist, *Heterosigma akashiwo*, that code for two hypothetical proteins that are homologous to each other [31–33]. Interestingly, their sequences show association with the geographic origins of the isolates, while the rest of mtDNA did not show clear isolation-by-distance, demonstrating that the phylogenetic studies based on different segments of *H*. *akashiwo* mtDNA may yield completely different insights. These observations pose a simple but important question: is the traditional choice of gene markers from mtDNA, which have been widely adopted for organisms belonging to different kingdoms, suitable for phylogeographic or population genetic studies?

To answer this question, we surveyed mtDNA sequences of different organisms, with a particular focus on their intraspecific variations and distribution of heterogeneities over different regions. We found that the extent of the sequence variations differed widely depending on both the organism and the regions of mtDNA. The limitations of the usage of traditional phylogenetic markers are discussed in detail. In addition, we attempted to identify an mtDNA segment that can be adopted as an ‘approximate marker’ for mtDNA-based phylogenetic study. We adopted two multiple sequence alignment (MSA) strategies for the phylogenetic reconstructions to test how the choice of the step affects the final output of the entire analyses.

## Results and discussion

### Literature survey for the usage of phylogenetic mtDNA-based markers

**Fig 1:**
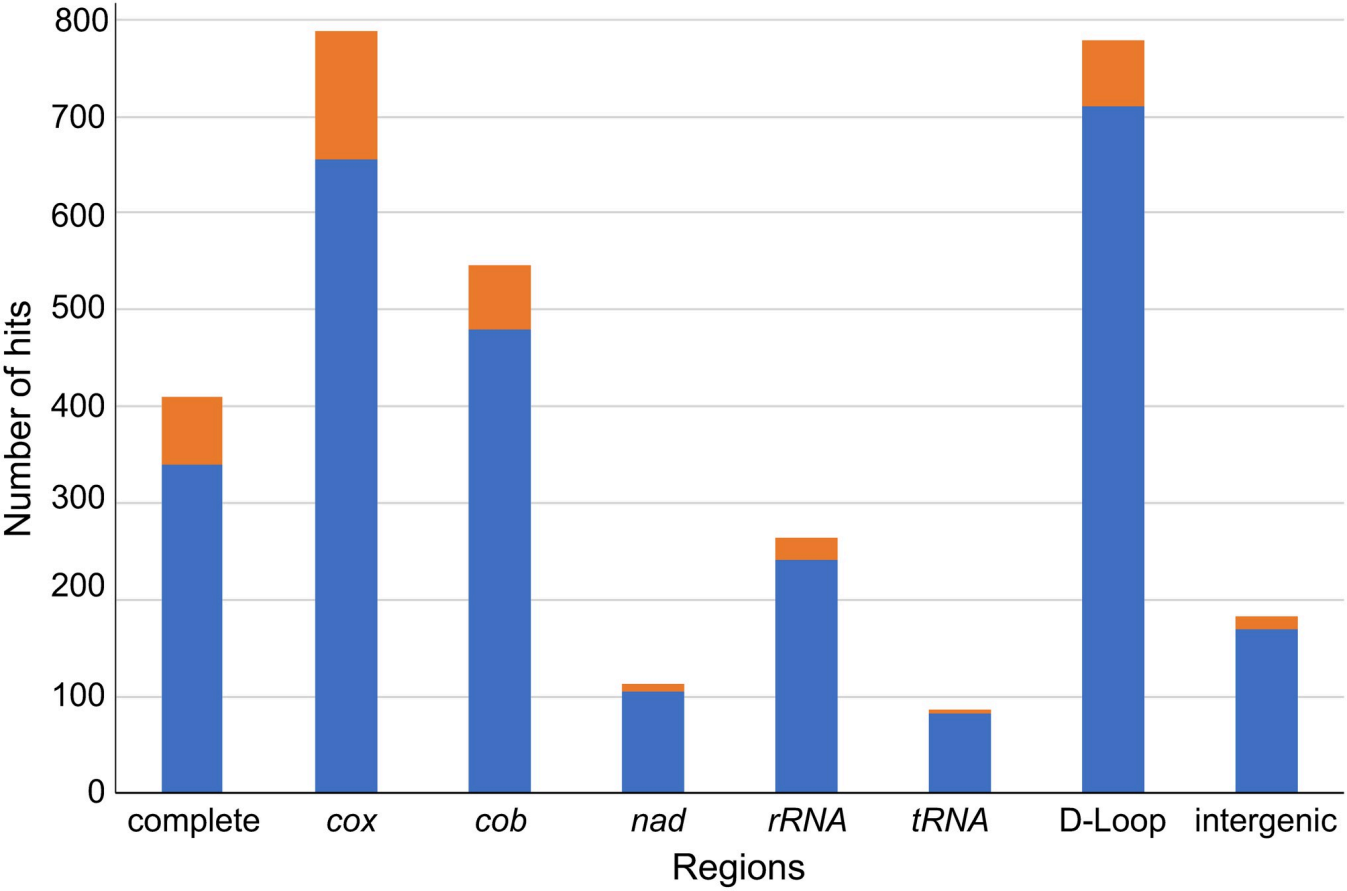
Number of literature hits by a PubMed search using various keywords related to different regions on mtDNA as of July 2021. The number of publications dated 2018 to 2021 are shown in orange, and the ones older than this period are shown in blue. Hit numbers for the intergenic region (IGR) do not include those for D-loop. The gene name abbreviations are as follows: Cytochrome c oxidase subunits, *cox*; cytochrome b, cob; NADH dehydrogenase subunit, *nad*.

We first surveyed PubMed to understand the trends in the usage of mtDNA-based markers for phylogenetic and population structure studies (Fig 1). Based on the search using different Boolean strings, the number of hits for a search for phylogeographic and population structure studies for non-human organisms using mitochondria-derived markers was 4,310 for the entire period and 541 from July 2018 to July 2021. The number of studies that contained the terms D-loop, *cox*, or *cob* were particularly plentiful, suggesting that these genes/segments have been frequently adopted as markers for this purpose. The number of studies based on full-length mtDNA published to date was ~12.9% of the total number of studies. The number of studies based on full-length mtDNA during the past three years was 17.1% of those based on all markers published during the entire period, suggesting that usage of complete mtDNA for phylogenetic or population structure studies became more common recently. At the same time, the studies based on D-loop, *cox*, and *cob* during the past three years were 12.7%, 24.5%, and 12.2% of the total number of studies conducted during the same period, respectively, suggesting that these markers were still widely used. *cox* was frequently used for studies on invertebrates, plants, and fungi, and D-loop was most often used for vertebrate studies (S1 Table).

### Skewed distributions of sequence heterogeneities on mtDNA in various species

Previously, our group demonstrated that two particularly variable protein-coding regions of *H*. *akashiwo* showed a strong association with their geographic origins, while other parts of mtDNA did not [31–33]. This may demonstrate that, at least in certain species, analyses on different parts of the mtDNA sequences yield varied phylogenetic insights. To test if this is generally the case, we sampled mtDNA sequences from a variety of taxa, obtained phylogenetic insights based on various parts of the mtDNA sequences, and observed the consistency of the results. To this end, we selected datasets for several organisms that consist of (1) complete mtDNA sequences with no ambiguity (i.e., N, W, M, R, etc., instead of A, T, G, and C), (2) at least 25 independent strains/individuals from the National Center for Biotechnology Information GenBank database for statistical analysis, and (3) sequences published with information on their geographical origin. Twelve organisms, including *Homo sapiens* and *Saccharomyces cerevisiae*, met the criteria. Because the usage of full-length mtDNA has been the norm for *H*. *sapiens* and *S*. *cerevisiae* for a substantial period [34–36], we finally chose ten datasets: *Apodemus agrarius* (striped field mouse [37]), *Camelus bacterianus* (domestic camel [38]), *Canis lupus* (gray wolf [39]), *Capra hircus* (domestic goat [40]), *Fusarium culmorum* (fungal plant pathogen causing seedling rot, foot rot, and ear blight to a wide range of monocots and dicots [41]), *F*. *graminearum* (fungal plant pathogen causing fusarium head blight on wheat, barley, and rice and ear rot on maize [41]), *Gallus* (domestic red junglefowl [42]), *H*. *akashiwo* (a protist, causative species of harmful algal bloom [31–33]), *Pan troglodytes* (chimpanzee [43]), and *Ursus arctos* (brown bear [28,29]). Such datasets were not available from the plant kingdom.

**Fig 2:**
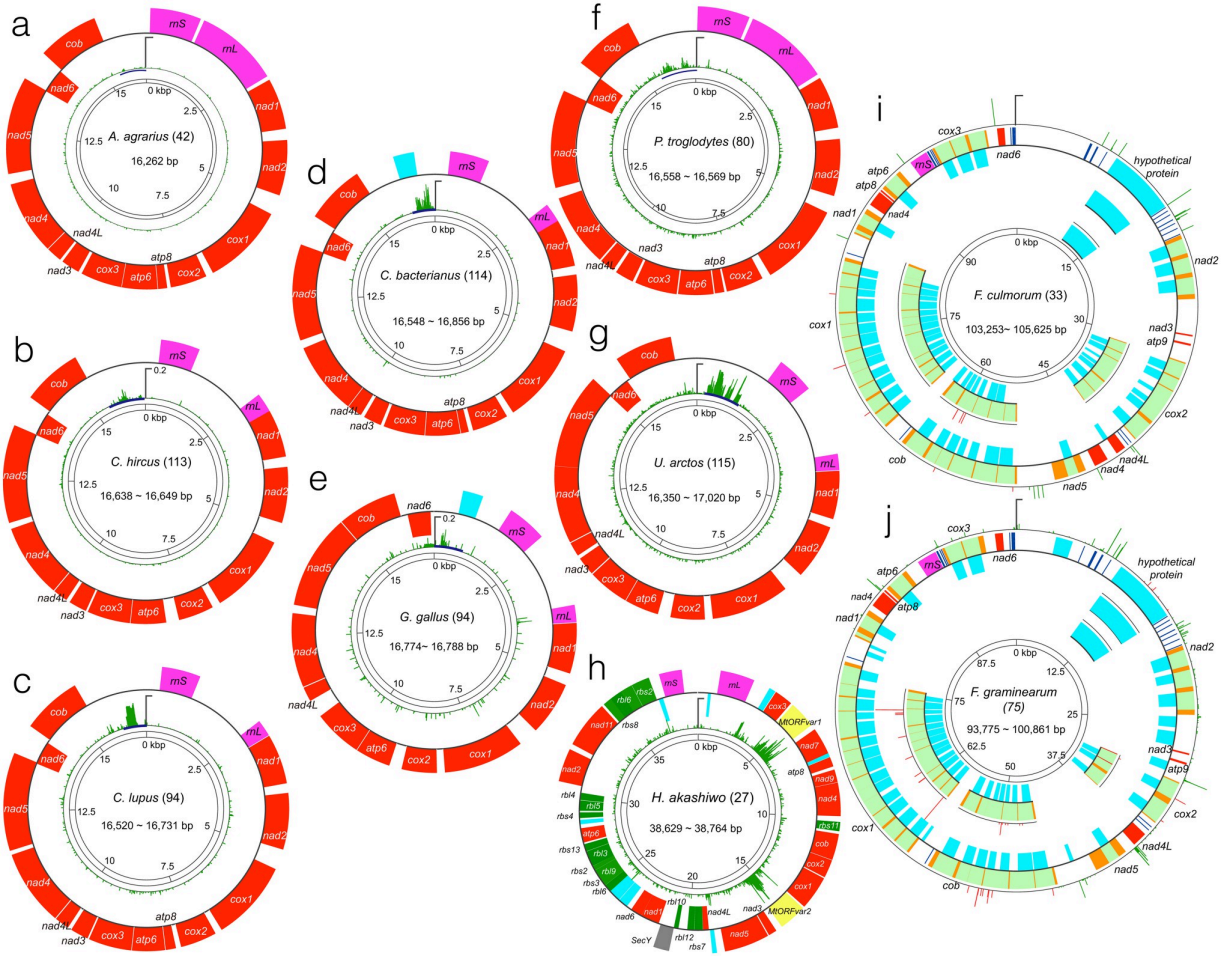
Genetic components (colored arcs) and sequence heterogeneities (green and red flairs) of the analyzed mtDNAs. Genes with different predicted functions are color-coded as follows: Respiratory genes, red; ribosomal subunit proteins, blue; SecY-independent transporter (SecY), grey; ribosomal RNA subunits, green; hypothetical proteins, cyan; MtORFvar1 and MtORFvar2, yellow; and ribosomal RNAs, pink. Introns and exons of respiratory genes are color-coded with pale green and orange, respectively, and tRNA-coding sequences are shown as dark blue bars in I) *F*. *culmorum* and J) *F*. *graminearum*. Genes coded by positive strands and negative strands in A-H are shown outside and inside arcs, while all of the ORFs in two fungal species (I and J) are coded by positive strands, and ORFs that are coded by the introns are shown as inside arcs. The gene name abbreviations are as follows: Ribosomal RNA large subunit, *rnL*; ribosomal RNA small subunit, *rnS*; cytochrome c oxidase subunit, *cox*; NADH dehydrogenase subunit, *nad*; ATP synthase F0 subunit, *atp*; ribosomal protein small subunit, *rbs*; ribosomal protein large subunit, *rbl*; and cytochrome b, *cob*. Scales for nucleotide positions are represented as grids and are numbered clockwise. The sequence heterogeneities among all sequences analyzed for the organisms are shown in green flair, while the heterogeneities among sequences with exon/intron organization variants and truncated versions of hypothetical proteins in *F*. *culmorum* and *F*. *graminearum* are shown in red. The scales for heterogeneities correspond to H = 0.5 unless otherwise noted.

First, we surveyed the distribution of sequence heterogeneities over the entire mtDNA of the organisms. Vertebrate species possess a small number of genes that lack introns in a largely conserved order arranged on compact genomes [6,7]. Their sequence heterogeneities were observed to be particularly concentrated in D-loops in all analyzed species, while other parts of the mtDNA, including *cox* and *cob*, had rather uniform heterogeneity distribution at much lower levels (Fig 2A–2G). Maximum heterogeneities observed in D-loops varies among the animal species; that of *A*. *argarius*, *G*. *gallus*, and *C*. *hircus* are particularly small, *C*. *bacterianus* is intermediate, and *C*. *lupus* and *U*. *arctos* are maximal. Protist mtDNAs are known to exhibit highly variable architectures, sometimes composed of multiple chromosomes, both circular and linear [18,22,44]. *H*. *akashiwo* possesses ~39 kbp circular mtDNA that codes for 17 respiratory genes, 16 ribosomal proteins, 2 rRNA subunits, 1 transporter protein, and 10 conserved hypothetical proteins (Fig 2H [31–33]). Previous studies revealed that two of the hypothetical proteins code for homologous proteins [31–33]. In this study, analysis of the newly sequenced mtDNA of 12 strains combined with the previously analyzed dataset further confirmed that these two protein-coding sequences, hypervariable open reading frames 1 and 2 (*MtORFvar1* and 2), expressed particularly high sequence heterogeneities (Fig 2H [31–33]). An intergenic region between large ribosomal RNA and a hypothetical protein, termed the ‘intergenic variable region’ in previous studies [31–33], is also particularly highly variable (Fig 2H), while the sequences of the region did not express isolation-by-distance [31–33].

Two fungal species, *F*. *culmorum* and *F*. *graminearum*, possess mtDNA with larger sizes, and all genes are coded by the same strand (Fig 2I and 2J). In many fungal species, mitogenes contain a variable number of groups I and II large introns that code for mobile endonuclease ORFs [17,41]. In these species, *cox1*, cox2, and *cob* in particular express intraspecific variations in exon/intron numbers; also, a couple of truncated versions of a hypothetical protein are found in the isolates. While expressing major variations in length and exon/intron configuration for these genes among strains, the sequence variations within each variant are notably smaller compared to mtDNA from other kingdoms (Fig 2I and 2J). In addition, sequence heterogeneities were particularly high in protein non-coding regions, both in intergenic regions and introns, suggesting that protein sequences are highly conserved among the different isolates (Fig 2I and 2J).

Overall, sequence heterogeneity distribution patterns were significantly different over the analyzed kingdoms, and commonly selected marker genes, *cox1*, *cob* and *nads*, are not particularly variable.

### Correlation of phylogenies between partial and entire mtDNA sequences in different species and effects of the choice of multiple sequence alignment strategies on the phylogenies

**Fig 3:**
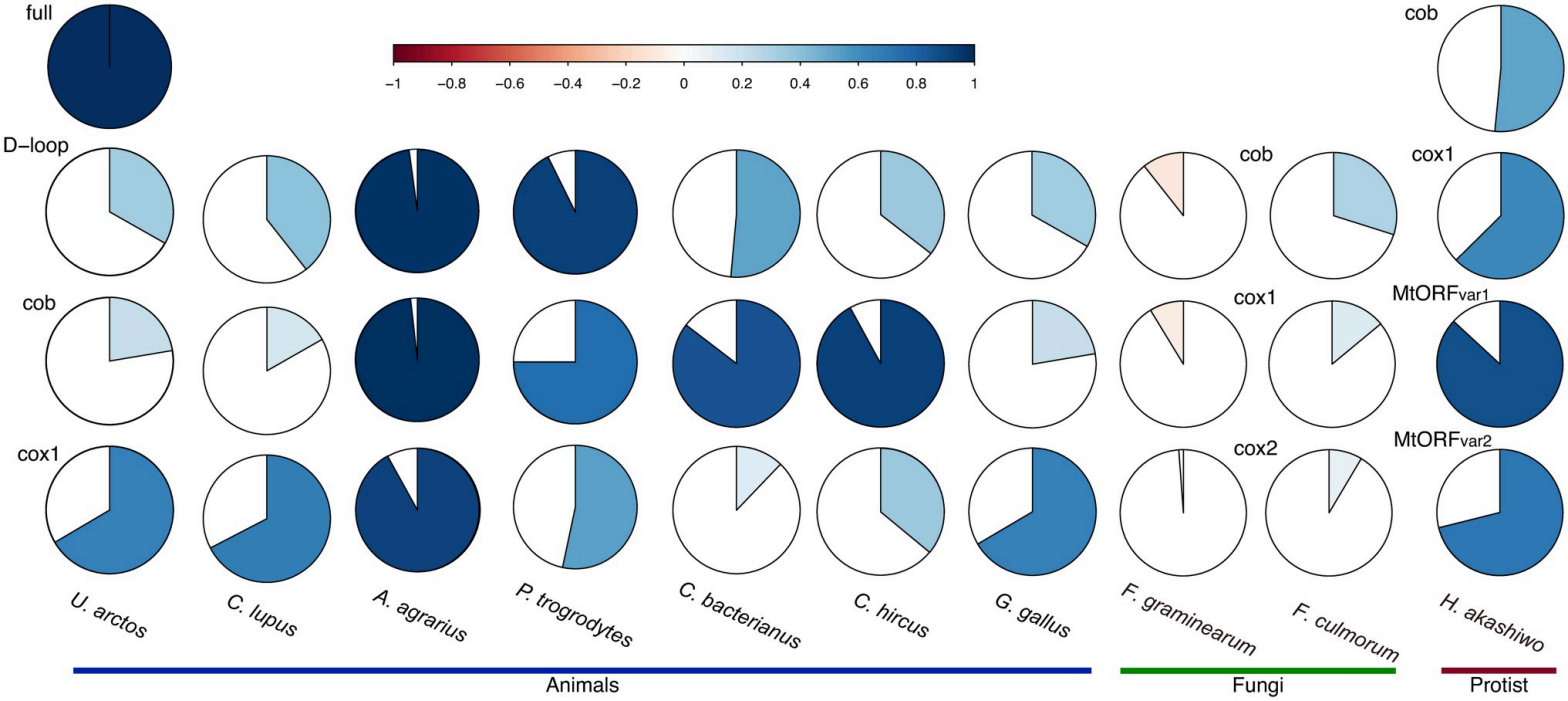
Correlations between the PRANK-aligned MSA-based phylogenies of full-length mtDNA sequences and those of indicated genes/segments on mtDNA. Note that a complete match between two data sets, for example, the correlation between the *U*. *artcos* full-length mtDNA dataset to itself, yields the rightmost top chart (full), and the extent of correlation and ratio of the correlated combinations to all tested combinations are expressed by color (top bar) and chart.

Ideally, partial mtDNA sequences could be adopted as convenient phylogenetic markers when their phylogenies reproduce those of the entire mtDNA. To test if this is the case, we evaluated the relatedness between the phylogenies of each gene and entire mtDNA quantitatively. The extent of the correlation between the phylogenies based on the entire and different genes/segments of mtDNA varies depending on the species (Fig 3 and S1 Fig). Because several studies revealed that the choice of MSA methods and implementation affected the downstream analyses [45–48], we adopted two different MSA methods, PRANK followed by GUIDANCE2 (Fig 3) or MAFFT (S1 Fig), and compared the resulting phylogenetic reconstruction. For example, *A*. *agrarius* showed high correlations between trees based on the entire mtDNA and all of the tested partial mtDNA sequences in the results from both analyses. In contrast, *C*. *hircus*, *C*. *bacterianus*, *and G*. *gallus* showed relatively low correlations between all analyzed partial and entire mtDNA in both analyses, suggesting that none of the three adopted partial mtDNA sequences would reproduce the phylogeny of the entire mtDNA. It may be noteworthy that the topologies of the phylogenetic trees obtained from D-loop that accumulated sequence heterogeneities at the highest levels in the entire mtDNA of all of the tested animal species did not necessarily correlate well with those of the entire mtDNA sequences, demonstrating that the segment of mtDNA from animal species may not consistently reproduce the phylogeny of the entire mtDNA. Although the correlation between PRANK-based and MAFFT-based full-length mtDNA phylogenies was high (S1 Fig), the extent of the correlation of *cox1*, *cob*, or D-loop to the entire mtDNA deduced from either MAFFT and PRANK were significantly different. To date, several studies have demonstrated that the quality of the phylogenetic reconstructions based on GUIDANCE2-verified PRANK MSA prevails over those based on MAFFT-based MSA [45–47]. Our observation further underscored the importance of the choice of MSA package for downstream analyses. In cases of two fungal species, the phylogenies of all analyzed genes expressed moderate to low correlation with that of the entire mtDNA by both analyses (Fig 3 and S1 Fig). This may be because the strains of the species possess mtDNA consisting of different combinations of various genotypes of *cox1*, *cox2*, and hypothetical protein variants. Here, again, analyses adopting either gene as the representative marker may yield significantly different results compared to those based on the entire mtDNA. In the case of *H*. *akashiwo*, the sequence variations of all tested mitochondrial genes correlated to that of entire mtDNA at certain extents. In general, these data revealed that the phylogenies based on the full-length mtDNA and partial sequence of mtDNA do not necessarily correlate with each other, and the gene/segment that phylogenetically correlated best to the entire mtDNA differs depending on the species.

### Comparison of phylogenetic reconstructions based on entire and partial mtDNA sequences

To gain more detailed information regarding the similarities or discrepancies between the phylogenetic insights obtained from entire and partial mtDNA sequences, we compared the clustering pattern of the phylogenetic trees reconstructed by these analyses. We chose three organisms, *A*. *agrarius* (the closest correlation between all selected genes and the entire mtDNA is shown in Fig 3 [37]), *P*. *troglodytes* (the second-highest correlation is shown in Fig 3 [28,29]), and *H*. *akashiwo* (expressed correlation at intermediate levels [31–33]), and compared the results with previously published observations. For the comparison, the partial mtDNA whose phylogeny exhibited the highest correlation with that of the entire mtDNA (Fig 3) was chosen for each organism.

**Fig 4:**
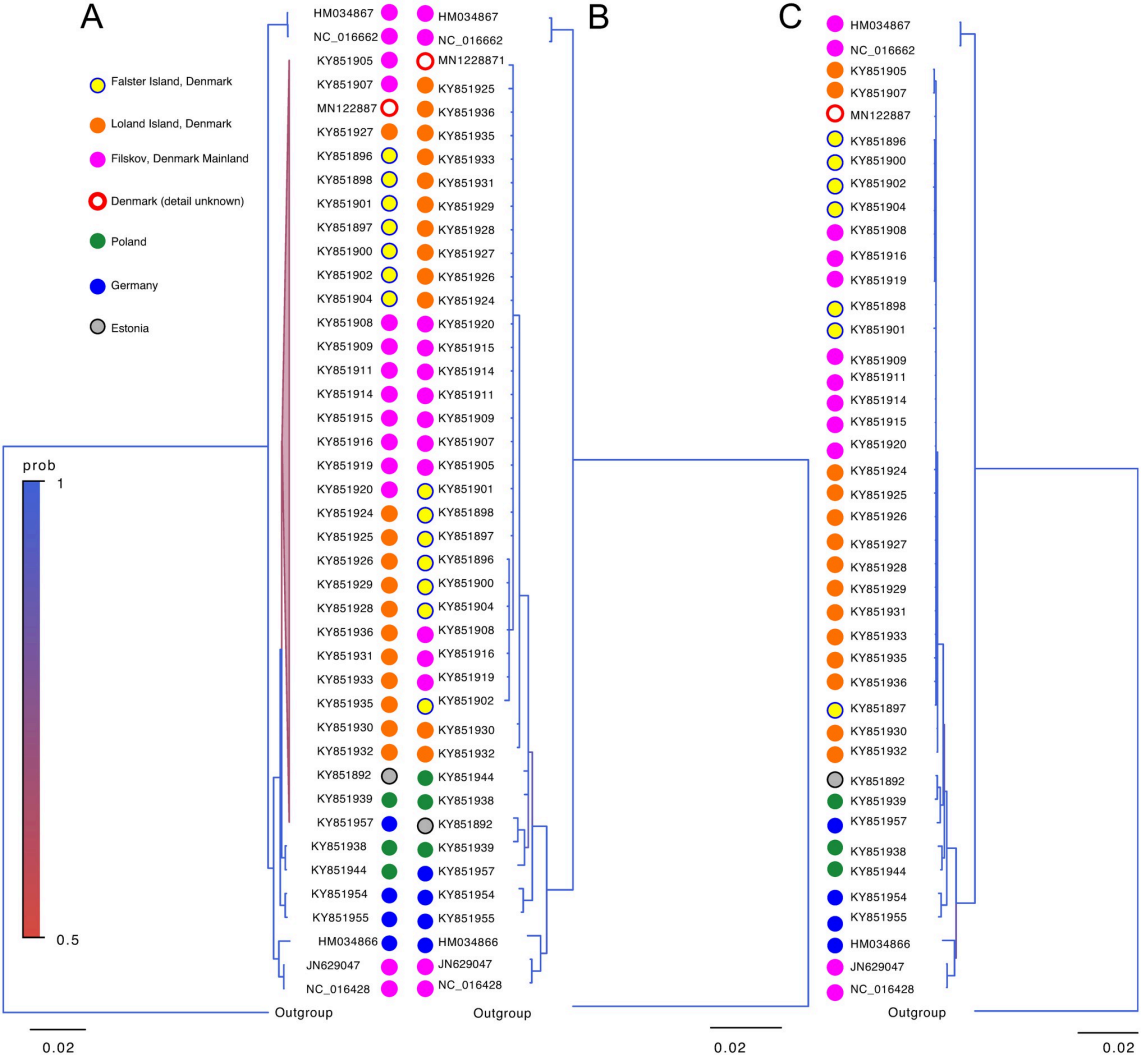
Phylogenetic divergence based on (A) full-length mtDNA and (B) *cob* and (C) contiguous sequence including *cob* to *rnS* of *A*. *agrarius*. The phylogenetic trees were reconstructed and rooted with *A*. *latronum* as an outgroup (NCBI accession HQ333256.1). Branches with a posterior probability of 0.5 to 1.0 are expressed in color as indicated. Each branch is color-coded by the geographic origin of the individuals, as indicated in (A).

Phylogenies of *A*. *agrarius* based on the entire mtDNA and *cob* sequences showed similar branching patterns (Fig 4). Most of the individuals from Denmark’s mainland and islands were grouped together and segregated from individuals who originated in mainland Europe, while two individuals from Denmark’s mainland clustered with individuals from Germany. While smaller datasets that are composed of sequences without any ambiguity were used for the analyses, the phylogenetic insight obtained here was consistent with the one previously published to show the colonization process of the species in Denmark [37].

In the case of *P*. *troglodytes*, while full-length mtDNA and cob phylogenies showed similar clustering patterns, the posterior probabilities at many branches are lower in cob phylogenies, resulting in phylogenetic reconstruction of lower resolution.

Finally, we conducted a phylogenetic reconstruction of full-length *H*. *akashiwo* mtDNA and *MtORFvar1* sequences (Fig 6). Previous phylogeographic studies with a smaller number of sequences revealed that *MtORFvar1* and -2 showed significant isolation-by-distance. The sequences of the isolates that were obtained from latitudinal regions higher than the previously defined geographic border, 42°N, formed a separate clade from others, with some exceptions [31–33]. In this study, when the entire mtDNA and *MtORFvar1* sequences were analyzed, most of the strains obtained from >42°N of the United States of America (USA) Atlantic, USA Pacific, and Europe areas were segregated from the strains obtained from other regions (Fig 6A and 6B). Notably, CCAP 934_8 and CCMP1595, isolated from Seattle, Washington, USA, and Rhode Island, USA, respectively, did not cluster with the >42°N clade (Fig 6A and 6B) in the current study. In addition, Haek9505-1 isolated from Tampa Bay, Florida, USA, was associated with the high latitude lineage as an exception, as previously observed [31–33]. In addition, as observed for *A*. *agrarius* and *U*. *arctos* datasets, analyses on the entire mtDNA of *H*. *akashiwo* yielded trees with higher posterior probabilities compared to those constructed based on the *MtORFvar1* analyzed here (Fig 6). These results demonstrate that, even when the phylogenies based on the partial mtDNA sequence and the entire mtDNA correlate well, at least for the datasets analyzed in this study, phylogenetic analyses based on high-quality, entire mtDNAs yielded results with higher resolutions and confidence compared to those based on partial sequences. In addition, the genetic distances estimated based on the entire and partial mtDNA are significantly different in *P*. *troglodytes* and *H*. *akashiwo*, while estimations based on the *A*. *agrarius* dataset were close to each other.

### To design a novel mtDNA-based marker that approximates the phylogeny based on the entire mtDNA

Our observations demonstrated that the traditional markers yield fewer phylogenetic insights compared to the entire mtDNA sequences, even for the dataset whose genetic distances among the sequences closely correlate to those of the entire mtDNA dataset. This may be because the longer sequences contain more substitution sites in the dataset and are thus more phylogenetically informative than shorter sequences. Alternatively, it may be because the entire mtDNA consists of both coding and non-coding sequences. The protein-coding sequences evolve under the pressure of functional constraints and thus yield different evolutionary patterns compared to the non-coding region. A segment may serve as a practical phylogenetic marker if it contains both coding and non-coding regions and the phylogenetic inferences of those resemble the one based on entire mtDNA. To identify such a segment, several different lengths of partial mtDNA sequences were selected and the concordance between the phylogenetic trees based on those datasets and the entire mtDNA were compared. To obtain convenient marker candidates that closely reproduce the phylogenetic inferences based on mtDNA in a reproducible manner, we attempted (1) to identify the contiguous and shortest segment of mtDNA that best reproduces the phylogeny of the entire mtDNA, and (2) to test if the randomly selected, smaller datasets of the short sequences reproduce the phylogeny of the entire mtDNA.

**Fig 5:**
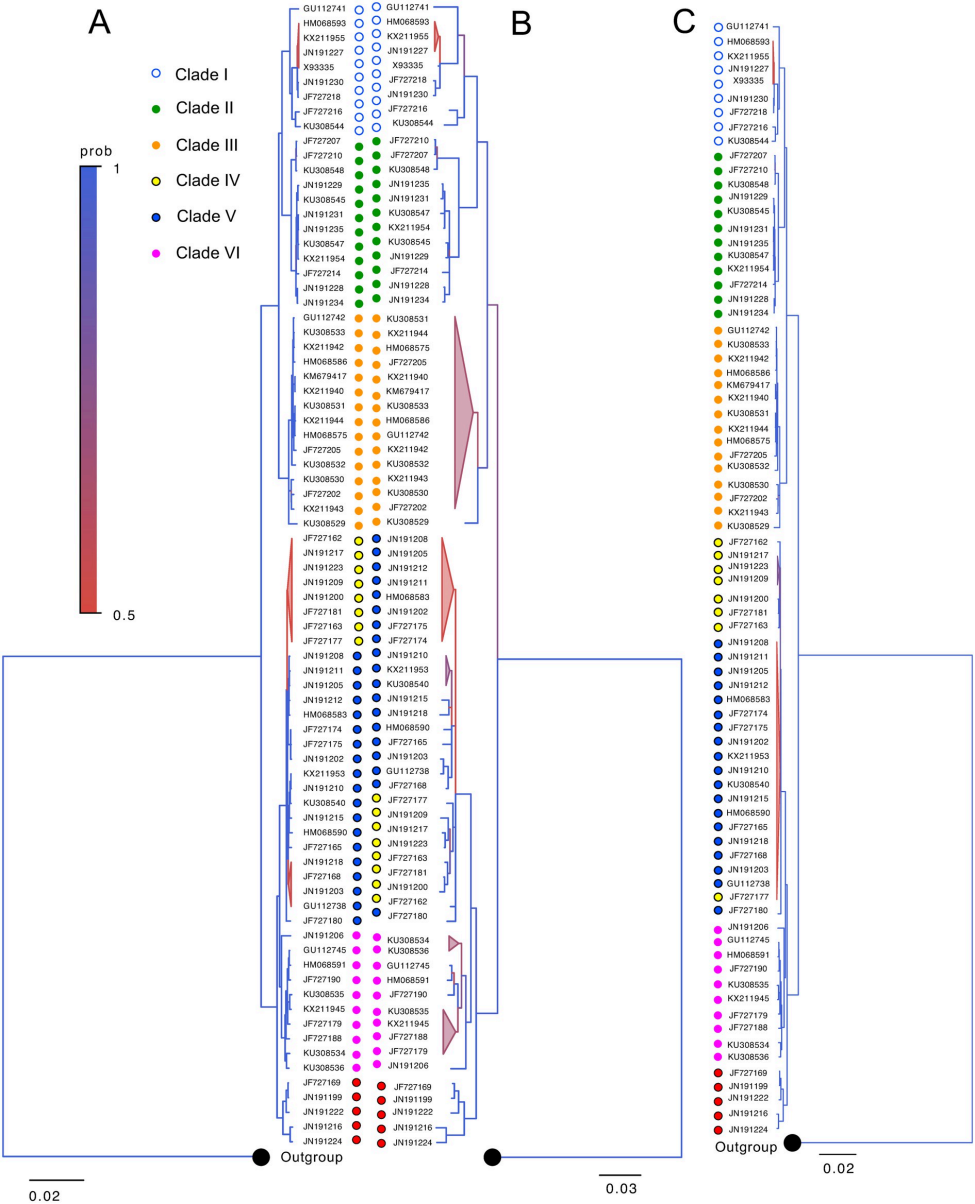
Phylogenetic divergence based on (A) full-length mtDNA and (B) *cob* and (C) contiguous sequence including *cob* to *rnS* of *P*. *troglodytes*. The phylogenetic trees were reconstructed and rooted with *Gorilla gorilla gorilla* an outgroup (NCBI accession NC 011120.1). Branches with a posterior probability of 0.5 to 1.0 are expressed in color as indicated. Each branch is color-coded by the clades in (A) as indicated.

**Fig 6:**
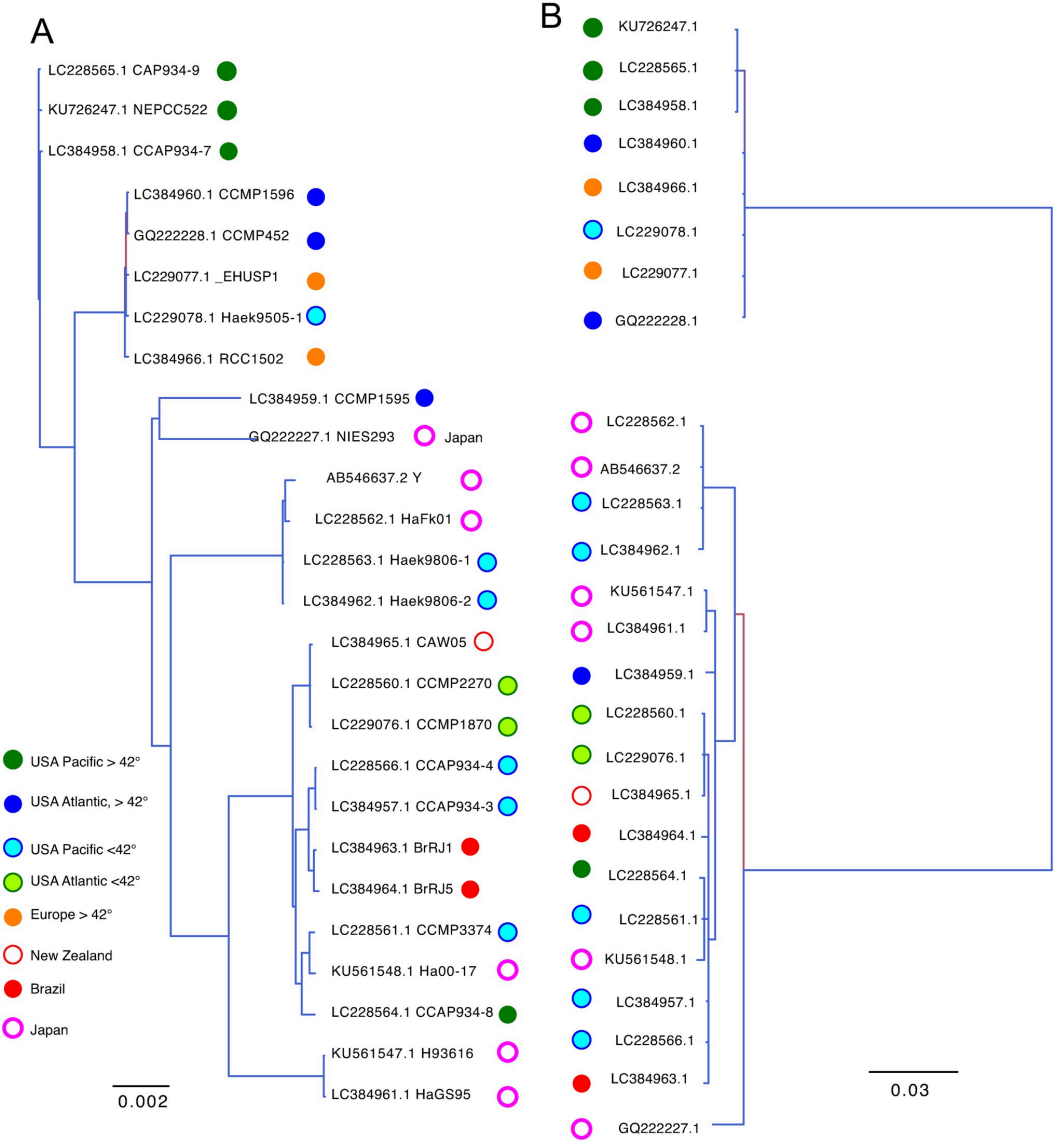
Comparison of the phylogenetic divergence of (A) *H*. *akashiwo* full-length mtDNA and (B) MtORFvar1. Branches with a posterior probability of 0.5 to 1.0 are expressed in color as indicated. Each branch is color-coded by the geographic origin of the isolate, as indicated in (A).

**Fig 7:**
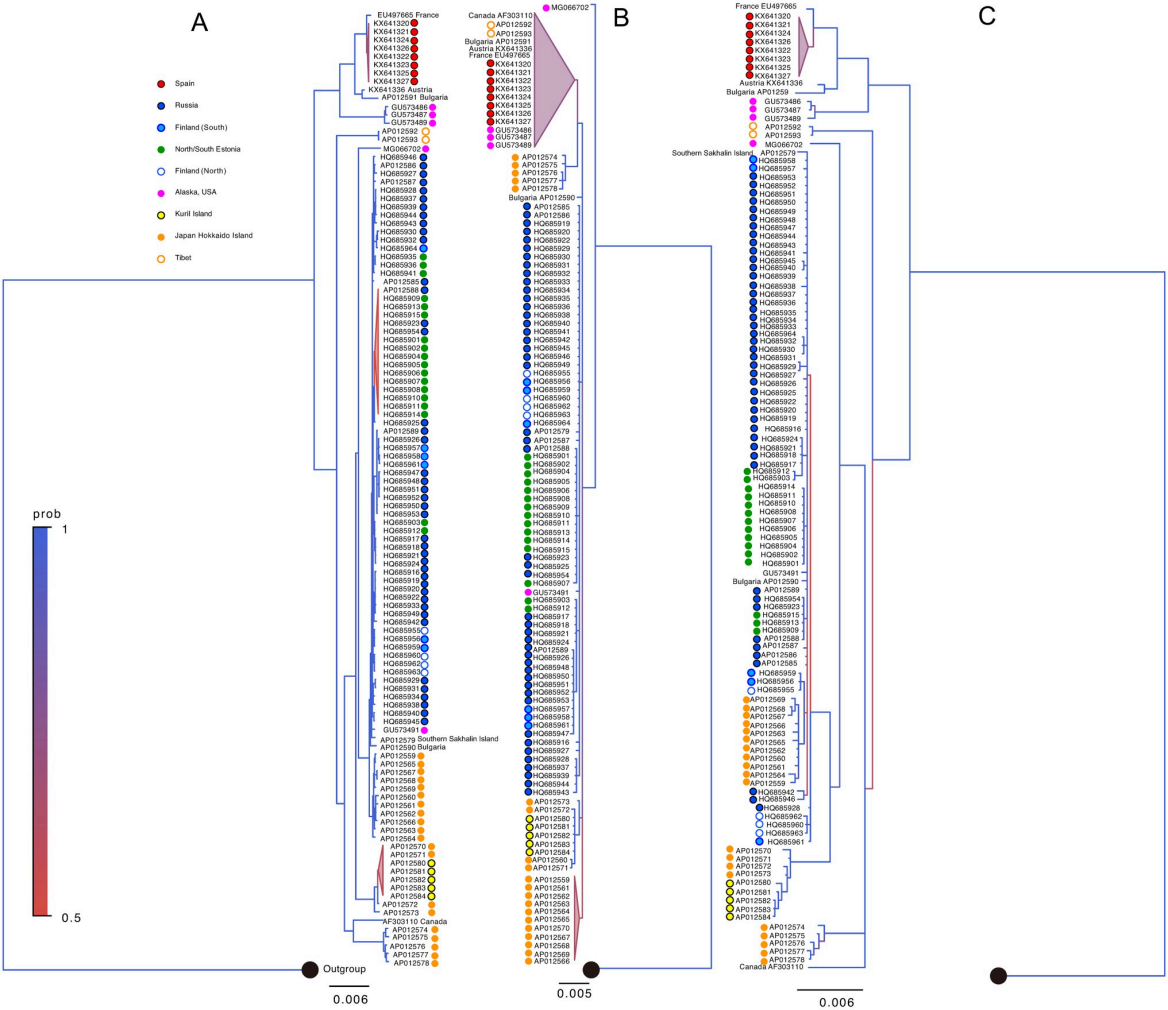
Phylogenetic divergence of (A) full-length mtDNA and (B) cox1, and (C) contiguous sequence including *cob* to *rnS* of *U*. *arctos*. The phylogenetic trees were reconstructed and rooted with *Ursus thibetanus ussuricus* as an outgroup (NCBI accession EF681884.1). Branches with a posterior probability of 0.5 to 1.0 are expressed in color as indicated. Each branch is color-coded by the geographic origin of the individuals, as indicated in (A).

**Fig 8:**
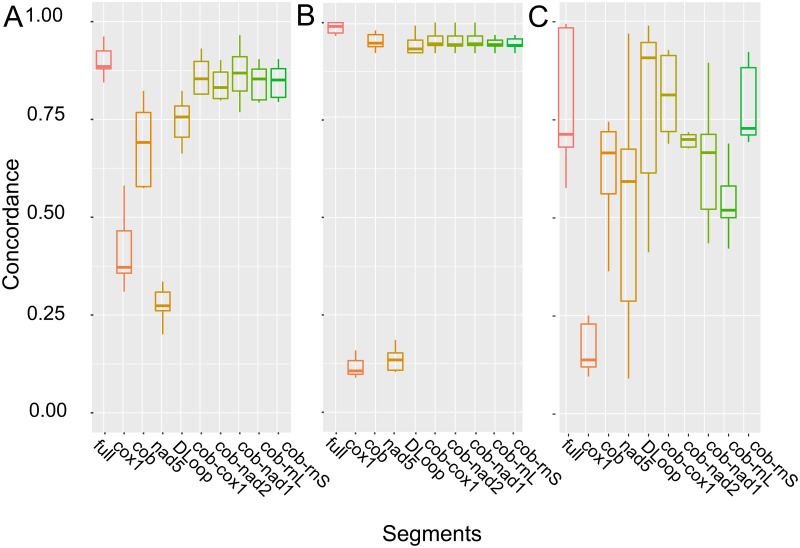
Concordances of the phylogenetic trees constructed based on full-length mtDNA and partial mtDNA sequences of (A) *A*. *agrarius*, (B) *P*. *troglodytes*, and (C) *U*. *arctos*. The phylogenetic trees of the complete and partial mtDNA of the randomly selected sequences of each organism were constructed, and the concordances of these trees to the one constructed on the entire dataset of complete mtDNA are indicated as box-and-whisker plots. *cob*-*cox*, *cob*-*nad*2, *cob*-*nad*1, *cob*-*rnL*, and *cob*-*rnS* are the contiguous segments spanning through the D-loop region and contain the entire sequences of *cob* and indicated genes at the terminals.

To this end, we selected three vertebrates, *A*. *agrarius* (Fig 4), *P*. *troglodytes* (Fig 5), and *U*. *arctos* (Fig 7), that showed clear clustering in the phylogenetic trees based on the entire mtDNA datasets, in which five out of six most ancestral branching were supported by >75% of posterior probabilities (Figs 4–7 and S2–S4 Figs). The mtDNA part whose phylogenetic reconstruction is most concordant with the one based on the entire mtDNA would be a useful phylogenetic marker. The analysis based on the single gene or D-loop showed consistently smaller concordance to the entire mtDNA compared to the analysis based on mtDNA segments containing both coding and non-coding sequences (Fig 8).

*Cob*, *cox*, and D-loop showed different levels of concordances to the entire mtDNA depending on the organisms, further demonstrating the risk of adopting the traditional markers. Next, we selected several contiguous parts of mtDNA that contain *cox*, *cob*, *nads*, *rnS*, *and rnL* on their terminals, and spanning through D-loop. Analyses based on *cob-rnS* showed significantly higher congruence to the results based on the complete mtDNA than *cob* or D-loop, and the congruence was comparable to those based on the longer sequences, such as *cob*-*cox1* (Fig 7). When the analyses were conducted using the MAFFT-aligned MSA, overall congruences of the partial mtDNA datasets were lower than the results obtained from PRANK-MSA and, particularly in *U*. *arctos*, *cob*-*rnS* showed significantly lower congruence to the full-length mtDNA than *cob*-*cox1* (S6 Fig). These results suggest that the usage of PRANK-GUIDANCE2-based MSA may allow simulation of the phylogenetic reconstruction obtained from the entire mtDNA by analyzing shorter partial sequences closely.

Further, the phylogenetic trees based on the entire mtDNA and *cob-rnS* segments showed substantially higher homology compared to the trees based on the entire mtDNA and an mtDNA gene (Figs 4A, 4C, 5A, 5C, 7A and 7C). It may be noteworthy that the genetic distance estimated from the *cob*-*rnS* datasets tended to be closer to those estimated from full-length mtDNA than the single gene or D-loop of the organisms (Figs 4, 5 and 7).

The higher correlations between trees based on full-length mtDNA and *cob*-*rnS* compared to the other mtDNA segments were also confirmed for two mammalian organisms, *C*. *bacterianus* and *C*. *lupus*, whose branching in mtDNA phylogenetic trees was supported by low posterior probabilities (S2 and S3 Figs). In the case of *C*. *hircus*, neither of the analyzed mtDNA segments showed high correlation with the phylogeny based on the entire mtDNA (S4 Fig). This may be, at least partially, because the phylogeny based on the entire mtDNA shows ambiguous clustering among most of the analyzed individuals.

These results suggest that phylogenetic analyses based on the segments may provide ‘approximate markers’ for mtDNA. Because *cob* and *rnS* coding sequences are highly conserved, an amplicon-seq-based approach can be adopted to simultaneously obtain the sequences of several individuals at a feasible cost. Designing a set of primers for amplicon-seq that targets the region followed by massively parallel sequencing adopting multiple barcoding may provide a convenient approach to utilize the segments as an approximate marker. Because the cob-rnS segment is 3.5 to 4.2 kb compared to ~16 kbp full-length mtDNA, the sequencing costs will be greatly suppressed by adopting this approach.

Whether utilization of mtDNA-based markers, either entire or partial, for a phylogenetic approach is biologically appropriate remains a debatable issue. The appropriateness of the usage of partial mtDNA sequences for the approach is one point. Our study demonstrated the importance of the choice of the mtDNA segments to reproduce the phylogeny of the entire mtDNA depending on the organisms, and proposed the segments that may be utilized as approximate markers for animals. Further, whether the sequence variation of the entire mtDNAs, which are only inherited maternally in most organisms and evolve faster than the nuclear genome, properly represents the genetic variation of the tested individuals has yet to be evaluated. For example, mtDNA is maternally inherited in most species, and therefore it may give a biased view of population history [49]. The importance of an integrative approach based on the nuclear genome and mtDNA to estimate the species history has been suggested recently [50]. The several phylogenetic studies that have been conducted to date based on both mitochondrial genes and the nuclear internal transcribed spacer (e.g., [51–53]) may contribute to obtaining an integrative evaluation of both mitochondrial and nuclear molecular evolutions. Utilization of our approximate marker as well as a genome-wide approach, such as RAD-seq, may be useful to incorporate both nuclear and mtDNA variations so as to decipher the evolutionary history of a species in an integrative manner. Such approaches can also be adopted to understand the differential divergence of nuclear and mtDNA sequences in a variety of organisms. For non-vertebrate organisms, including invertebrates, plants, fungi, and protists, the partial mtDNA markers that show congruence with the entire mtDNA should be determined empirically.

### Conclusions

Our findings may underscore the importance of the marker genes/segments choice on mtDNA to obtain proper information regarding the intraspecific genetic variations to analyze their phylogeny and population structure.

Our observations suggested that the choice of mitogene for phylogeographic or population genetic studies affected the results in a significant manner. This is a reasonable yet frequently disregarded premise. Due to the selection pressure on the function of the products, protein-coding sequences tend to be less divergent, resulting in smaller genetic distances and obscure clustering for phylogenetic analysis. In particular, the use of traditional marker sequences, whose products possess vital functions for the organism, should be considered with great care. Selection of a partial segment based on the comparison among entire mtDNA sequences of several individuals, as well as preliminary phylogenetic analysis based on a small number of samples, may improve the quality of the study. For such studies for mammalian, the segment cob-rnS, length of 3.5 ~ 4.2 kb, may serve as an excellent approximate marker.

## Materials and methods

### Literature search

The MEDLINE database was searched using the PubMed search engine to obtain published articles regarding mtDNA genetic variation, with a particular focus on phylogeographic and population genetics. Because usage of the complete mtDNA sequence for phylogenetic and population structure analysis was already the norm for humans in 2006 [34], these studies were excluded. Specifically, we used the Boolean search string “(mitochondrial AND (DNA or genome)) AND ((phylogeography [MeSH Terms]) OR (genetic, population[MeSH Terms])) NOT human,” then further narrowed the search with the following strings: for tRNA, (transfer RNA) OR (trna); for ribosomal RNA, (ribosomal RNA) OR (rRNA); for cytochrome c oxidase subunits, (cytochrome c oxidase) OR cox OR COI OR COII OR COIII OR CO1; for cytochrome b, (cytochrome b) OR (apocytochrome b) OR cob; for NADH dehydrogenase subunits, (NADH dehydrogenase) OR (nad); for D-loop, (“D Loop”) OR (“control region”); for intergenic regions, ((“intergenic region”) OR (“non-coding region”)) NOT ((“D loop”) OR (“control region”)); and for complete mtDNA, complete. The strings were selected to optimize the coverage of MeSH vocabularies.

### Datasets

The dataset consisting of complete and unambiguous mtDNA sequences of >25 strains/individuals from the same taxonomic ID (organisms) with information available on their geographic origin was selected for this study. As a result of the process, we finally chose 10 datasets: *Apodemus agrarius* (striped field mouse, 42 individuals), *Camelus bacterianus* (domestic camel, 114 individuals), *Capra hircus* (domestic goat, 112 strains), *Canis lupus* (gray wolf, 31 individuals), *Gallus* (domestic red junglefowl, 96 individuals), *Pan troglodytes* (chimpanzee, 80 individuals), and *Ursus arctos* (brown bear, 115 individuals), *H*. *akashiwo* (a protist, causative species of harmful algal bloom, 26 isolates), *Fusarium culmorum* (fungal plant pathogen causing seedling rot, foot rot, and ear blight to a wide range of monocots and dicots, 33 isolates), and *F*. *graminearum* (fungal plant pathogen causing Fusarium head blight on wheat, barley, and rice and ear rot on maize, 75 isolates). An appropriate dataset was not available from the plant kingdom.

### Preparation for the analyses

Because all of the sequences analyzed in this study were circular, we first aligned the sequences to be analyzed and redefined the start position of each genome at an identical position. The gene coding sequences (CDSs) on the genomes were re-annotated using MFannot https://megasun.bch.umontreal.ca/cgi-bin/dev_mfa/mfannotInterface.pl using the translation table for the specific taxa, and the CDSs were excised using the EMBOSS package (http://emboss.sourceforge.net).

### Sequence heterogeneity analysis

*To analyze the sequence heterogeneity*, *the entire mtDNA sequences were aligned by the MAFFT package*. The extent of variation at each aligned nucleotide position, heterogeneity (H), was calculated as

$$\text{H}=1-\Sigma {\text{p}}_{\text{i}}{}^{2},\;\left(\text{i}=\text{A},\;\text{T},\;\text{G},\;\text{C},\;\text{and}\;\text{gap}\right),$$

where p_i_ is the probability of the occurrence of i, and Σ stands for the summation over the five possibilities [54]. H of each segment was further processed using a sliding window approach with window size 10 and sliding by 5 for *U*. *arctos*, *C*. *lupus*, *C*. *hircus*, *G*. *gallus*, *C*. *bacterianus*, and *H*. *akashiwo*. The H and the open reading frames (ORFs) of the mtDNAs were visualized using ArcWithColor software that is bundled in the GenomeMatcher package [55] (http://www.ige.tohoku.ac.jp/joho/gmProject/gmhomeJP.html).

#### Phylogenetic reconstruction and the tree correlation and concordance analyses

For the phylogenetic studies, the PRANK and MAFFT package was utilized for the MSA.

The PRANK alignments were conducted using either the codon-conscious mode for protein-coding sequence or the nucleotide mode for the non-coding region [56]. Two *Fusarium* species possess protein-coding sequences that contain both introns and exons, thus the codon-conscious alignment was not adopted. The alignment was verified by the GUIDANCE2 package [57], and the nucleotides aligned at the scores >0.93 were extracted and subjected to further phylogenetic reconstructions using the iqtree [58] or MrBayes [59] packages. In the PRANK-Guidance-based analyses of the sequences of the entire mtDNA or more than one gene and intergenic regions, the aligned sequences were concatenated and subjected to downstream analyses. The MAFFT alignments were directly applied to the analysis by iqtree. The phylogeny trees were visualized using the Geneious package [60] or the FigTree package.

The correlations among the phylogenies based on the different datasets were evaluated using the corrplot function of R package ape (http://ape-package.ird.fr/ [61]). The concordance between the phylogenetic trees constructed based on the full-length mtDNA and partial sequences was evaluated using R package treespace (https://cran.r-project.org/web/packages/treespace/index.html [62]) “treeConcordance” functions. Because the latter analysis is designed to evaluate the concordance of the clade topologies of two phylogenetic trees, three organisms, *A*. *agrarius*, *P*. *troglodytes*, and *U*. *arctos*, that yielded phylogenies supported by high posterior probability values were subjected to the analyses. For analyzing the concordance of the partial mtDNA sequences to the entire mtDNA, 26 individuals of *A*. *agrarius*, 47 individuals of *P*. *troglodytes*, and 69 individuals of *U*. *arctos*, which correspond to ~60% of the available datasets, were randomly selected using the “rand” function of the perl package and subjected to the analyses.

## Supporting information

S1 Fig Correlations between MAFFT-aligned MSA-based phylogenies of the full-length mtDNA sequences and those of the indicated genes/segments on the mtDNA and PRANK-aligned full-length mtDNA MSA-based phylogenies. (TIF)

S2 Fig Phylogenetic divergence of *C*. *bacterianus* full-length mtDNA. (TIF)

S3 Fig Phylogenetic divergence of *C*. *lupus* full-length mtDNA. (TIF)

S4 Fig Phylogenetic divergence of *C*. *hircus* full-length mtDNA. (TIF)

S5 Fig Phylogenetic divergence of *G*. *gallus* full-length mtDNA. (TIF)

S6 Fig Concordances of the phylogenetic trees constructed based on the MAFFT-aligned full-length mtDNA and partial mtDNA sequences of (A) *A*. *agrarius*, (B) *P*. *troglodytes*, and (C) *U*. *arctos*. (TIF)

S7 Fig Correlations between the MAFFT-aligned MSA-based phylogenies of full-length mtDNA sequences and those of indicated genes/segments on mtDNA. (TIF)

S1 Table The number of phylogeographic and population genetics studies conducted for various organisms based on mitochondrial markers. (DOCX)
