# Supplementary figures and images for: Intraspecies variation of the mitochondrial genome: An evaluation for phylogenetic approaches based on the conventional choices of genes and segments on mitogenome
Source: PLoS One. 2022 Aug 18;17(8):e0273330. doi: 10.1371/journal.pone.0273330 (PMC9387813; doi:10.1371/journal.pone.0273330)

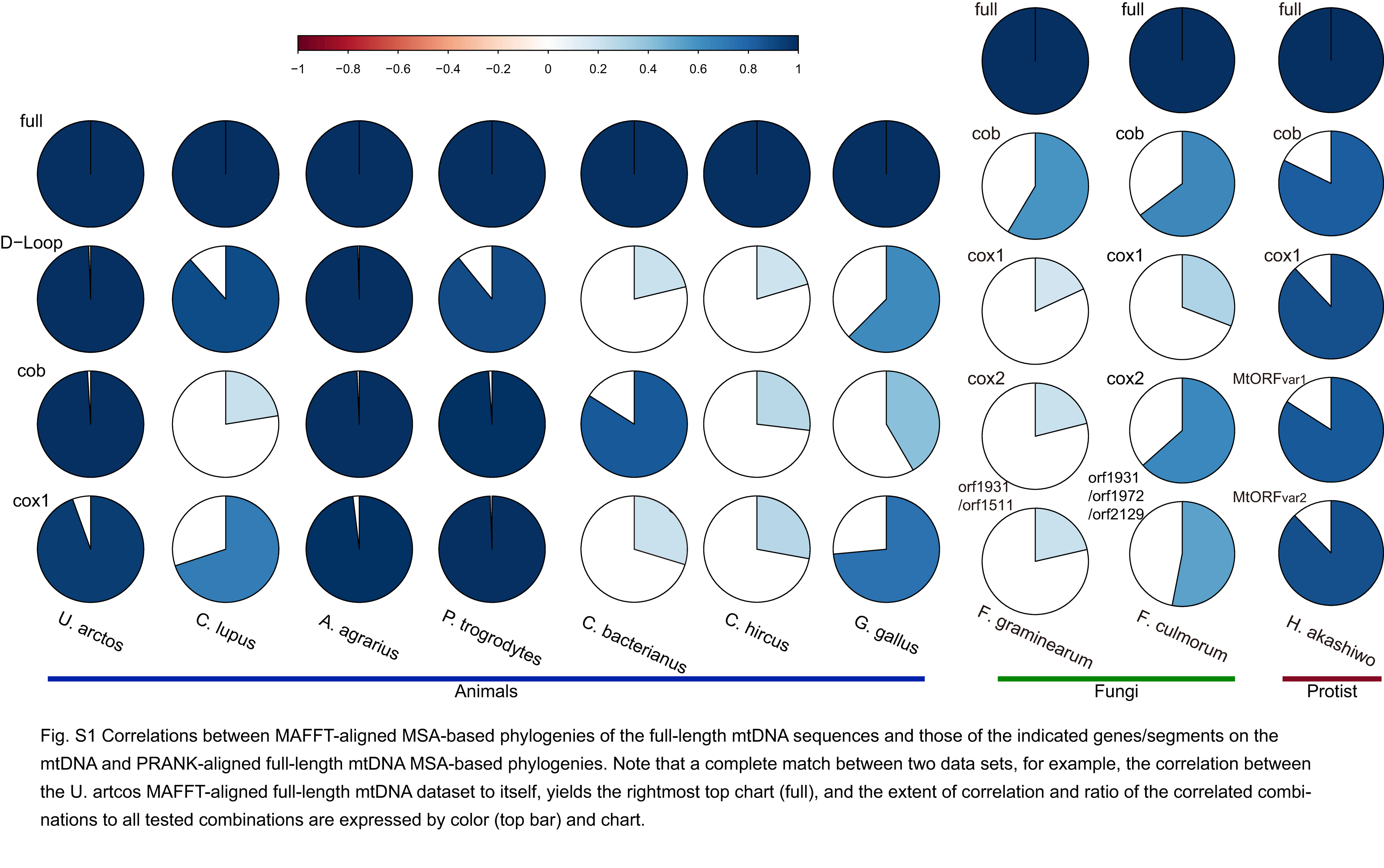

Supplement: S1 Fig — (TIF) [file pone.0273330.s001.tif]

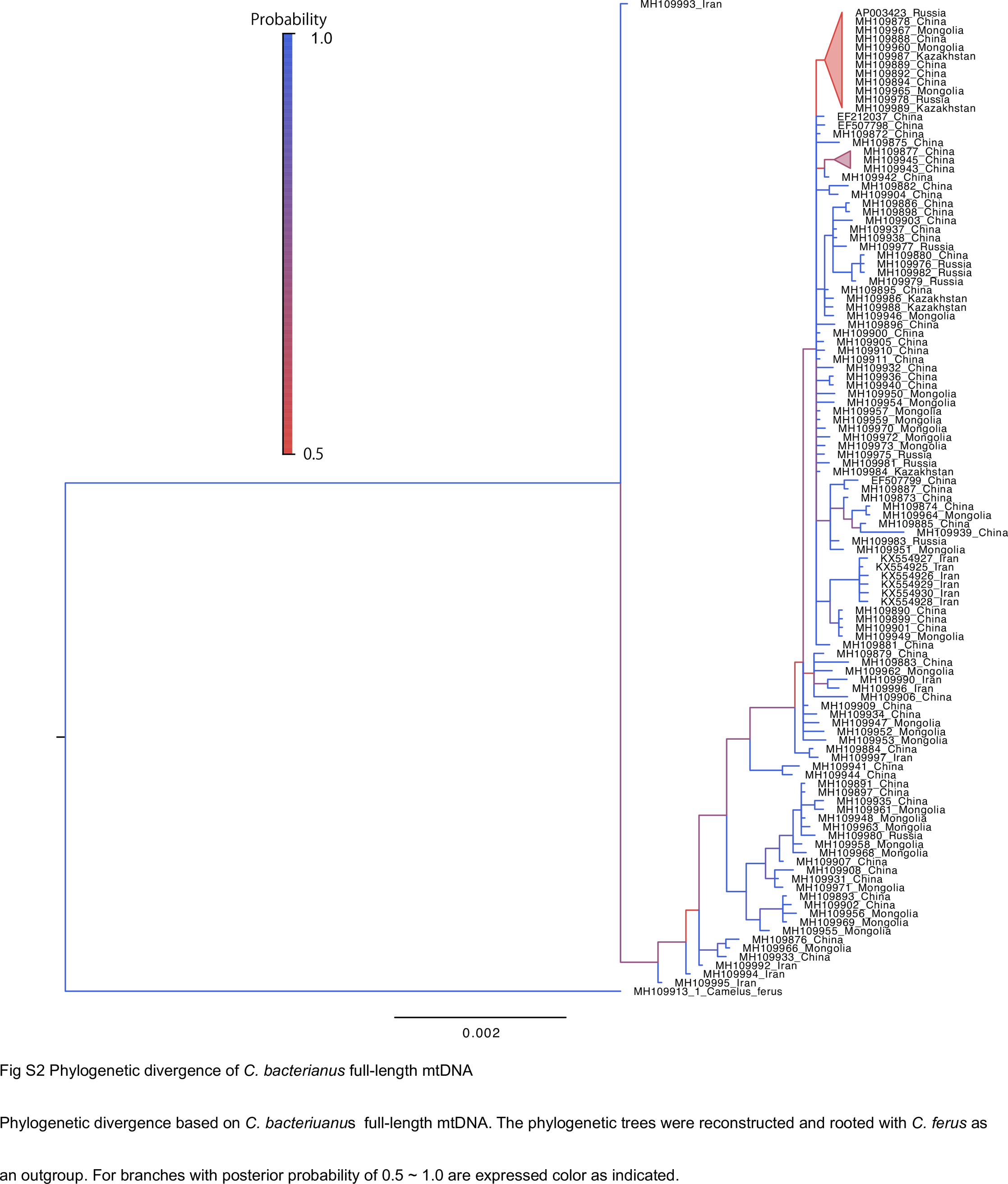

Supplement: S2 Fig — (TIF) [file pone.0273330.s002.tif]

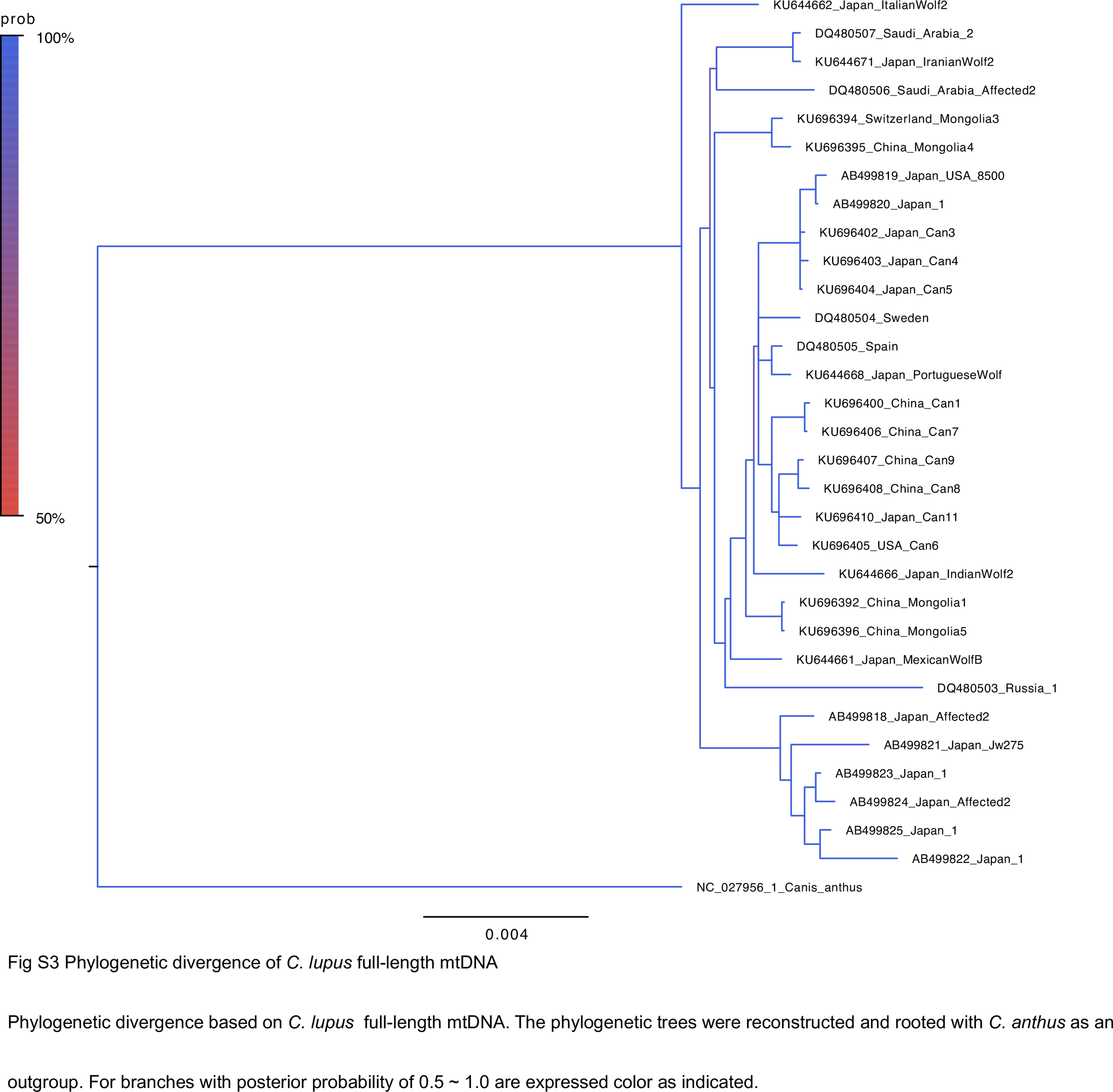

Supplement: S3 Fig — (TIF) [file pone.0273330.s003.tif]

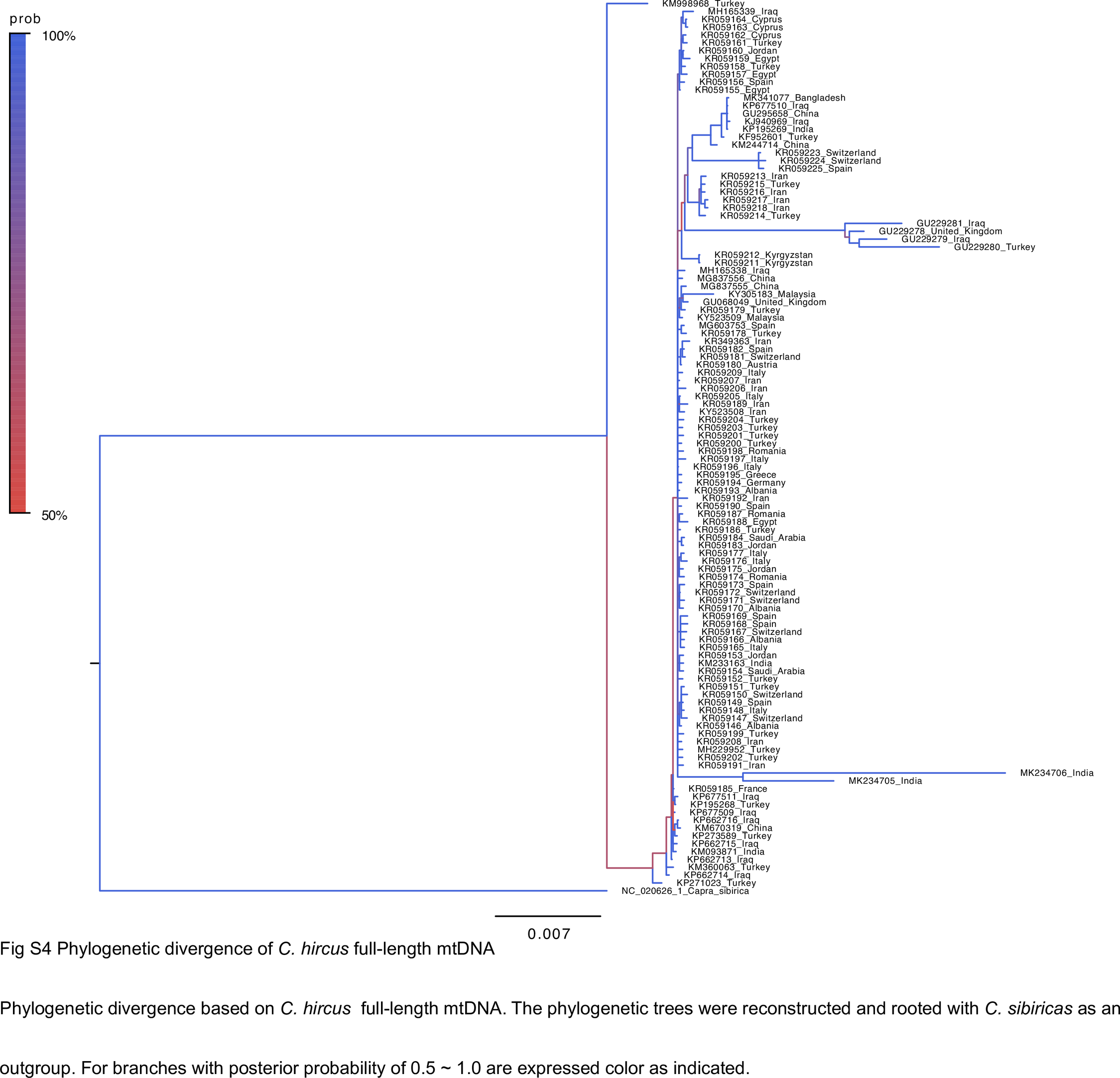

Supplement: S4 Fig — (TIF) [file pone.0273330.s004.tif]

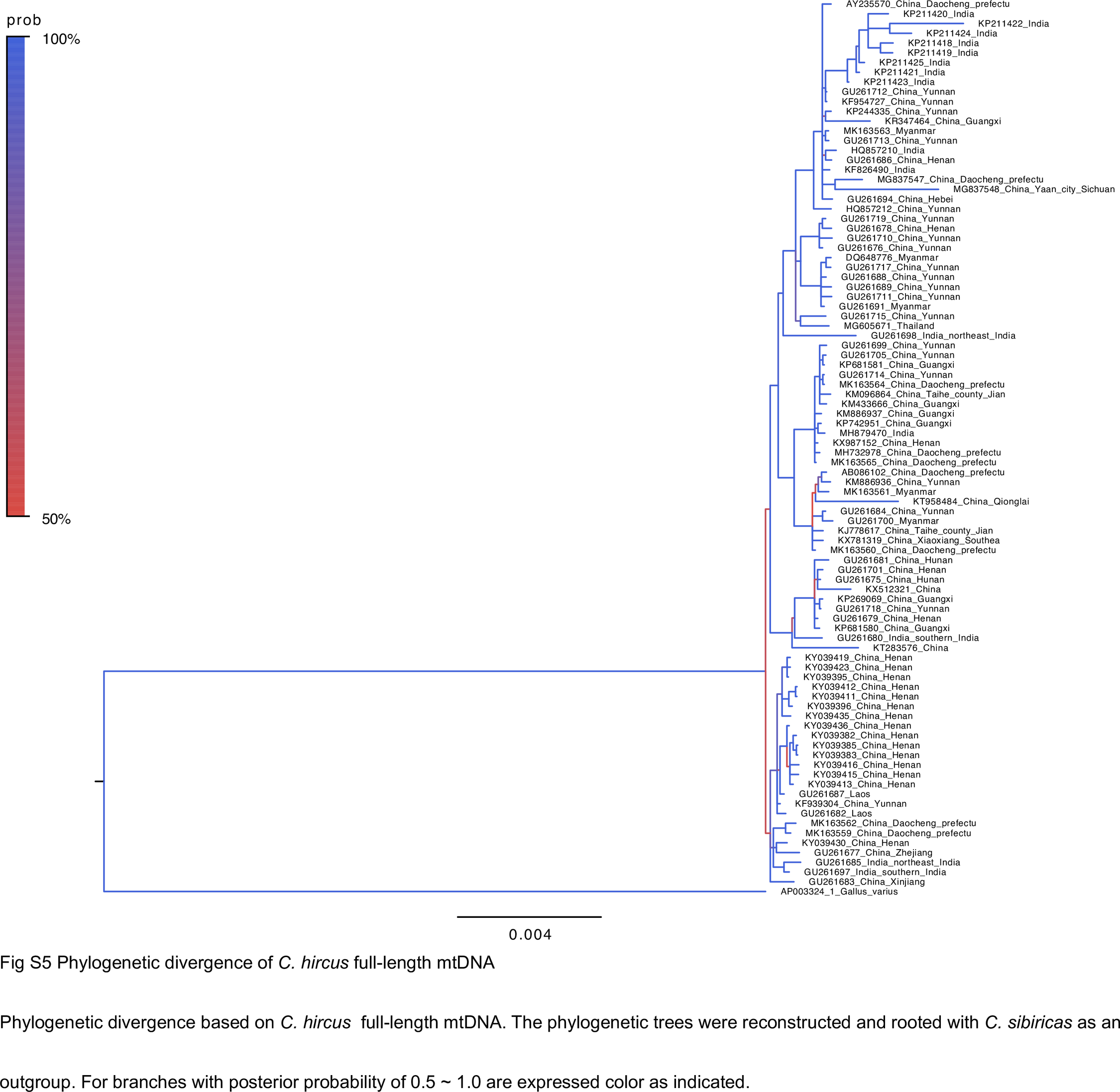

Supplement: S5 Fig — (TIF) [file pone.0273330.s005.tif]

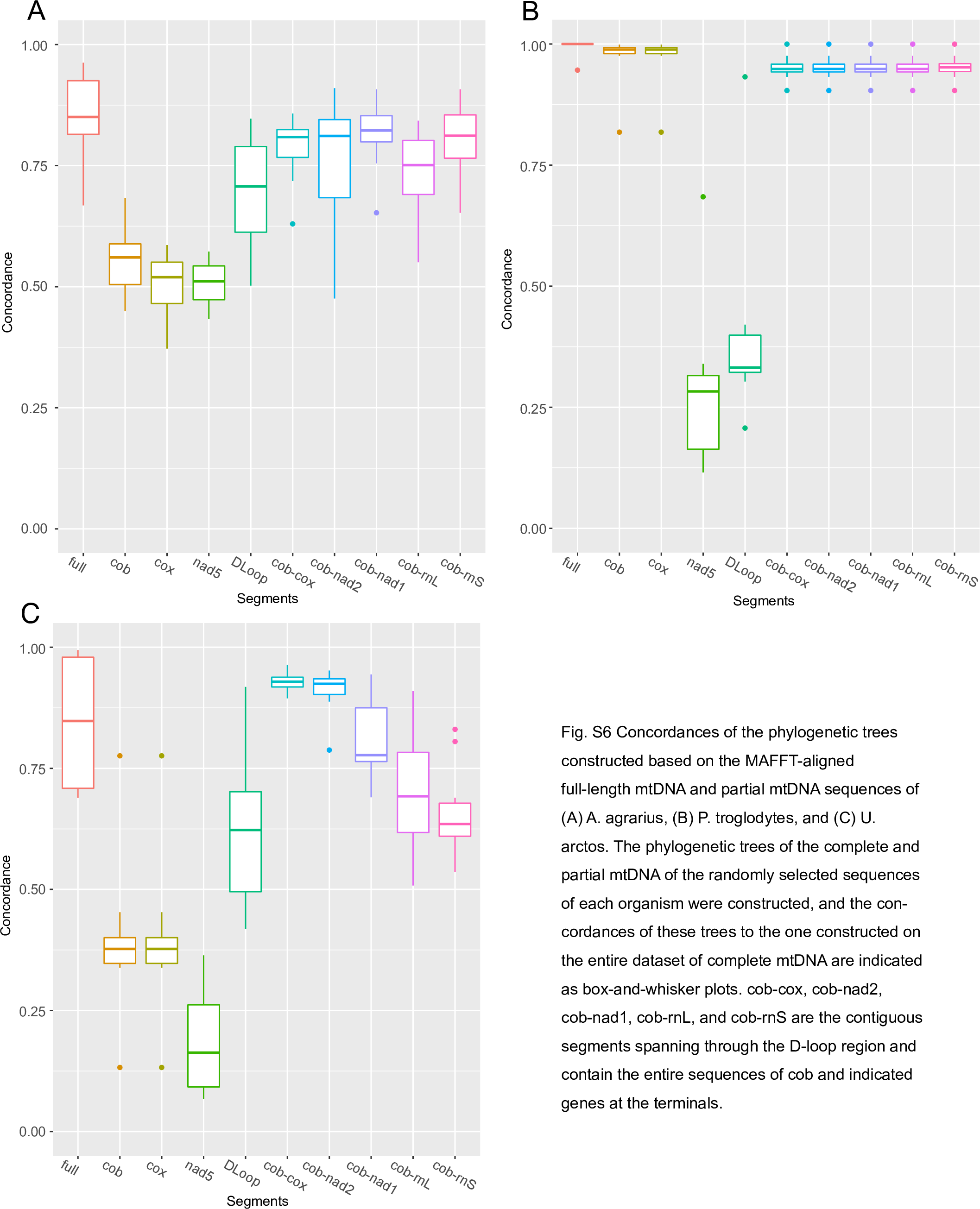

Supplement: S6 Fig — (TIF) [file pone.0273330.s006.tif]

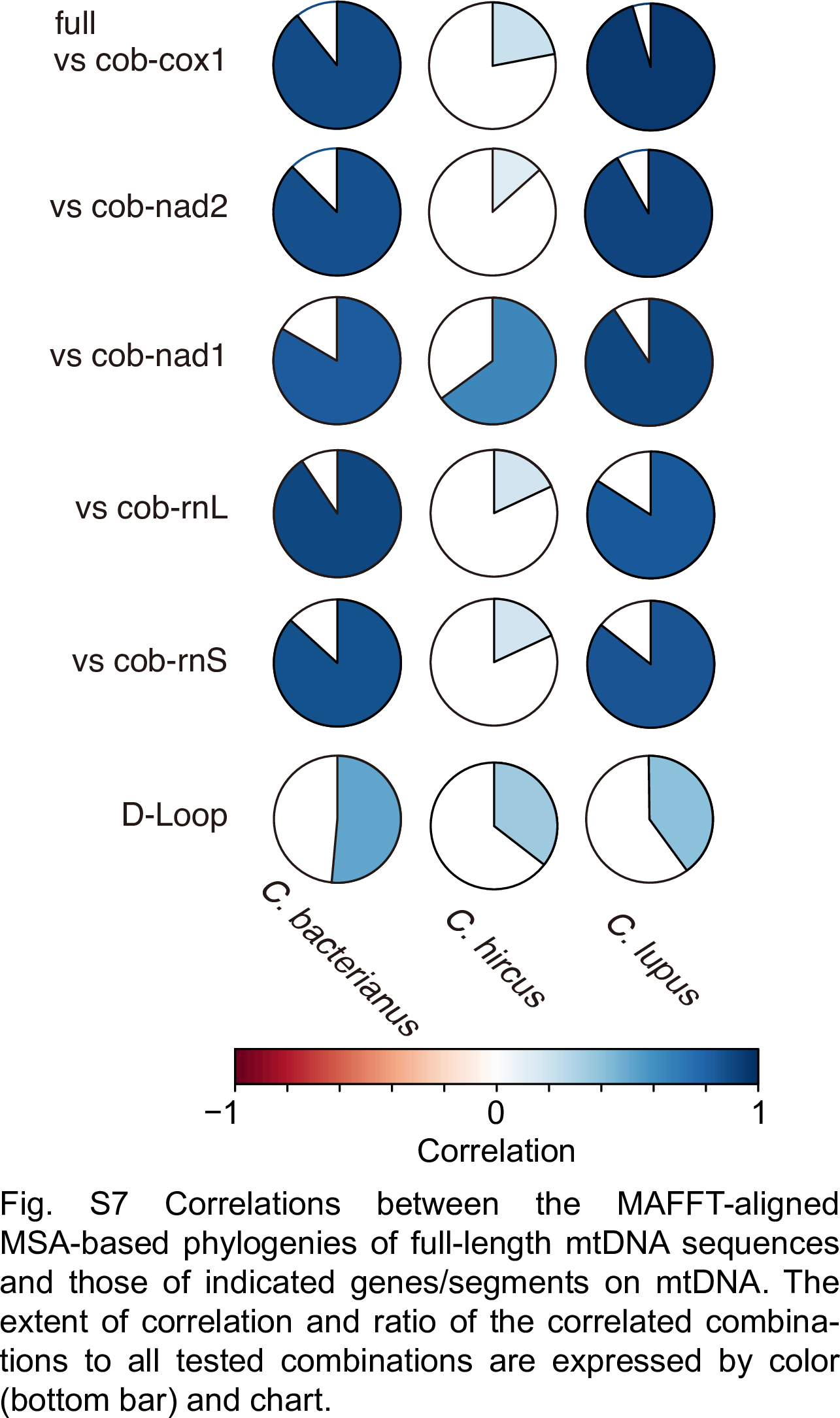

Supplement: S7 Fig — (TIF) [file pone.0273330.s007.tif]
