# Supplementary material for: Intraspecies variation of the mitochondrial genome: An evaluation for phylogenetic approaches based on the conventional choices of genes and segments on mitogenome
Source: PLoS One. 2022 Aug 18;17(8):e0273330. doi: 10.1371/journal.pone.0273330 (PMC9387813; doi:10.1371/journal.pone.0273330)
Supplement: S1 Table — (DOCX) [file pone.0273330.s008.docx]

Table S1 The numbers of the phylogeography and population genetics studies conducted for various organisms based on mitochondrial markers

| Markers | Studied organisms | | | |
| --- | --- | --- | --- | --- |
|  | vertebrates | invertebrates | plant | fungi |
| tRNA | 67 | 19 | 2 | 1 |
| *cox* | 196 | 627 | 110 | 37 |
| *cob* | 499 | 52 | 24 | 9 |
| *nad* | 77 | 42 | 8 | 2 |
| D-Loop | 726 | - | - | - |
| Intergenic^*^ | 80 | 88 | 37 | 11 |
| complete | 289 | 113 | 35 | 12 |
| rRNA | 119 | 0 | 24 | 19 |

^*^ Intergenic regions besides D-Loop.
